# Asparagine Synthetase in Cancer: Beyond Acute Lymphoblastic Leukemia

**DOI:** 10.3389/fonc.2019.01480

**Published:** 2020-01-09

**Authors:** Martina Chiu, Giuseppe Taurino, Massimiliano G. Bianchi, Michael S. Kilberg, Ovidio Bussolati

**Affiliations:** ^1^Laboratory of General Pathology, Department of Medicine and Surgery, University of Parma, Parma, Italy; ^2^Department of Biochemistry and Molecular Biology, University of Florida College of Medicine, Gainesville, FL, United States

**Keywords:** asparagine synthetase, acute lymphoblastic leukemia, asparagine, glutamine, cancer

## Abstract

Asparagine Synthetase (ASNS) catalyzes the synthesis of the non-essential amino acid asparagine (Asn) from aspartate (Asp) and glutamine (Gln). ASNS expression is highly regulated at the transcriptional level, being induced by both the Amino Acid Response (AAR) and the Unfolded Protein Response (UPR) pathways. Lack of ASNS protein expression is a hallmark of Acute Lymphoblastic Leukemia (ALL) blasts, which, therefore, are auxotrophic for Asn. This peculiarity is the rationale for the use of bacterial L-Asparaginase (ASNase) for ALL therapy, the first example of anti-cancer treatment targeting a tumor-specific metabolic feature. Other hematological and solid cancers express low levels of ASNS and, therefore, should also be Asn auxotrophs and ASNase sensitive. Conversely, in the last few years, several reports indicate that in some cancer types ASNS is overexpressed, promoting cell proliferation, chemoresistance, and a metastatic behavior. However, enhanced ASNS activity may constitute a metabolic vulnerability in selected cancer models, suggesting a variable and tumor-specific role of the enzyme in cancer. Recent evidence indicates that, beyond its canonical role in protein synthesis, Asn may have additional regulatory functions. These observations prompt a re-appreciation of ASNS activity in the biology of normal and cancer tissues, with particular attention to the fueling of Asn exchange between cancer cells and the tumor microenvironment.

## Introduction

Asparagine Synthetase (asparagine synthase (glutamine-hydrolysing) or glutamine-dependent asparagine synthetase, E.C. 6.3.5.4, ASNS) catalyzes the biosynthesis of asparagine (Asn) from aspartate through an ATP-dependent reaction that exploits the amido-N of glutamine (Gln) to form the amido group of Asn.

The human *ASNS* gene is located at chromosome 7q21.3 and is 35 kb long with 13 exons (1). The ASNS protein (561 aa) has two primary domains, termed the N- and C-terminal domains, and is expressed in many tissues, with a wide range of expression levels. Particularly high levels of expression are detected in the pancreas, brain, thyroid and testes, while the liver has low expression of ASNS. Several transcript varieties and putative isoforms of human ASNS have been described although information on their role in physiology and pathology is lacking.

ASNS deficiency (ASNSD, OMIM 615574) is an autosomal recessive, rare, severe disorder associated with congenital microcephaly, cognitive impairment, progressive cerebral atrophy, intractable seizures, and early death (2, 3). The prevalence of neurologic symptoms suggests that ASNS plays a unique role in brain development. Interestingly, plasma and cerebral spinal fluid Asn levels are lowered only in some of the patients tested thus far, preventing diagnosis on biochemical bases (4). For more detailed information on ASNS structure, enzymatic mechanism, and mutations associated with ASNSD, the reader is referred to recent reviews and original articles (5–7). In particular, the high-resolution crystal structure of human ASNS recently provided by Zhu et al. (7) indicates that the enzyme is composed of two domains, with the C-terminal synthetase domain more similar to ASNS in other organisms than the N-terminal glutaminase domain. The glutaminase domain has a topology similar to that of other amidotransferases and other conserved amino acid residues are present at the interface of the two domains where substrate recognition occurs. Also the amino acids in the synthetase site are for the most part conserved in human and bacterial ASNS.

## ASNS Regulation

Numerous studies have placed ASNS at the center of the cell response to amino acid deprivation and other forms of cellular stress [reviewed in (5, 8–10)]. Through transcriptional regulation, the *ASNS* gene is a target of two signaling pathways aimed to ensure cell survival under conditions of imbalanced amino acid availability, named the Amino Acid Response (AAR) (9), and of increased endoplasmic reticulum stress, the Unfolded Protein Response (UPR) (10). Through the activation of, respectively, the GCN2 and the PERK kinases, both these stress-response pathways converge on the phosphorylation of the α-subunit of the initiation factor eIF2, which provokes the attenuation of global protein synthesis and, at the same time, the preferential translation of a selected population of mRNAs, including the transcription factor ATF4. ATF4 is the major factor for *ASNS* induction, working as a trans-activator through the binding to an enhancer element within *ASNS* promoter (8). A very recent contribution (11) demonstrates that in Asn-depleted cancer cells a translational reprogramming, dependent on the increase of MAPK-interacting kinase 1 (MNK1) and eukaryotic translation initiation factor 4E (eIF4E), promotes enhanced ATF4 translation and, hence, *ASNS* expression. The role of other components of the UPR, such as IRE and ATF6, seems less important (12). However, *ASNS* transcription is also influenced by factors such as p53, which can serve as a negative regulator of the gene (13).

## Low ASNS Expression in Acute Lymphoblastic Leukemia: Old Observations and New Perspectives

Interest in the role of ASNS in cancer was initially due to the observation of low synthetic activity for Asn in malignant tissues (14, 15), which were, therefore, auxotrophic for Asn, thus accounting for sensitivity to bacterial L-asparaginases (ASNase). The widespread clinical use of ASNase in acute lymphoblastic leukemia (ALL) began in the 1970s and today is a cornerstone of multi-drug therapy for this hematological cancer (16, 17). Thus, ASNase represents the first, and until now uniquely successful, example of a therapeutic approach targeting a metabolic feature of a specific form of cancer. Moreover, the strict requirement for extracellular Asn of ALL blasts (and of some lymphoma models), due to low levels of ASNS protein expression, was the first example of a cancer-specific auxotrophy for a non-essential amino acid (18). More recently, other examples have been described in human cancers, such as the loss of argininosuccinate synthetase in hepatocellular carcinomas, metastatic melanomas, and other cancers, leading to auxotrophy for arginine (19), and the absence of glutamine synthetase expression in multiple myeloma (20) and oligodendroglioma (21), leading to Gln auxotrophy.

**Figure 1:**
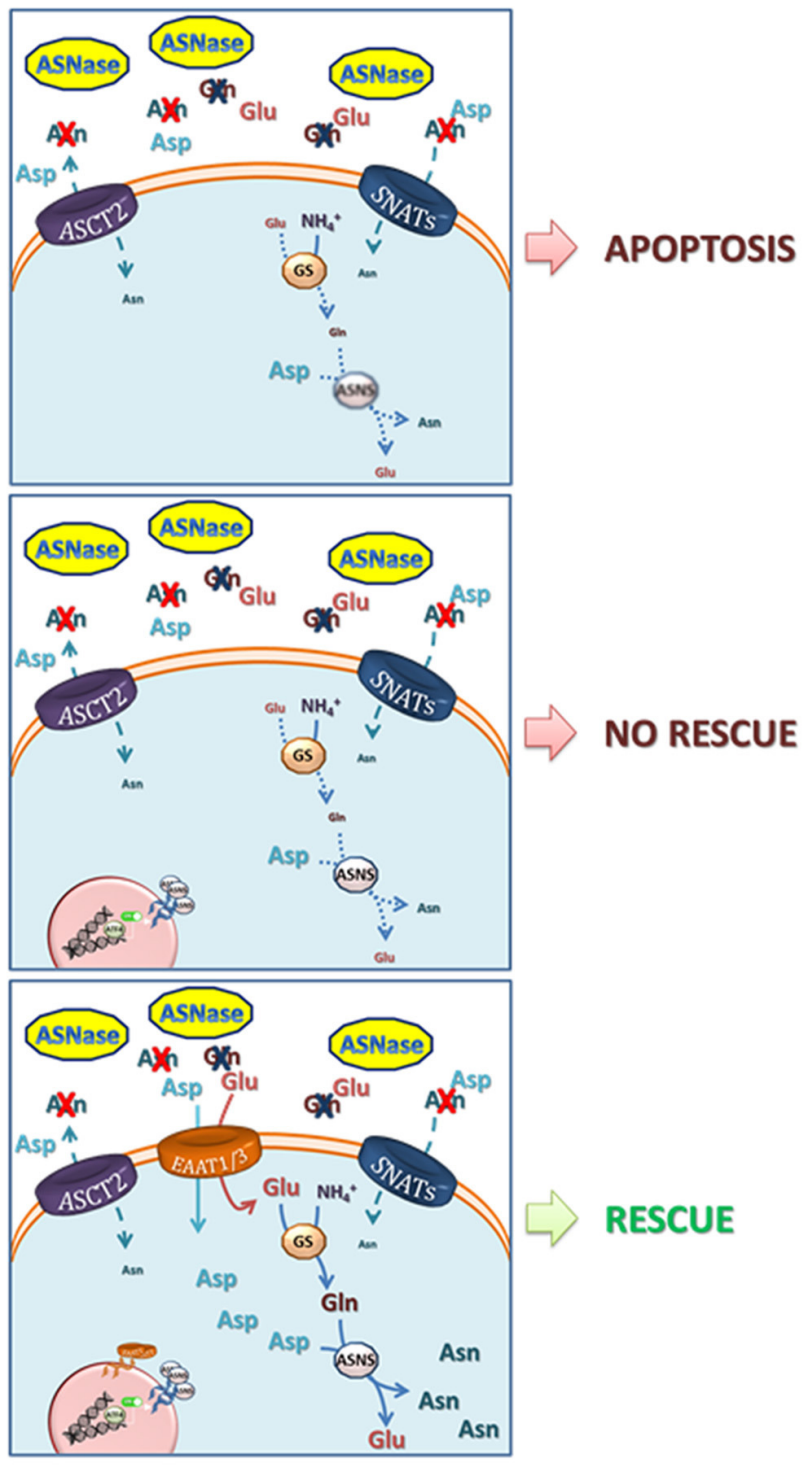
Mechanisms involved in resistance to L-asparaginase. Upper panel, ASNase catalyzes the hydrolysis of asparagine (Asn) into aspartate (Asp) and of glutamine (Gln) into glutamate (Glu), driving low-ASNS cells to cell death. Central panel, ASNS induction and increase in GS protein expression are not able to rescue ASNase-induced apoptotis due to poor availability of their substrates Asp and Glu. Lower panel, the overexpression of EAAT1 or EAAT3 anionic amino acid transporters provides Glu (for the synthesis of Gln, through Glutamine Syntethase) and Asp (27). Both Gln and Asp are needed for Asn synthesis via ASNS and for an effective cell rescue. The model is mainly based on data obtained with prostate cancer cells by Sun et al. (27) but it may apply to other low-ASNS cancers.

Given the low expression of ASNS, the incubation of ALL blasts with ASNase is rapidly followed by the fall of intracellular Asn and by a prolonged nutritional stress, which causes proliferative arrest and, eventually, apoptotic death of leukemia cells. In most normal and cancer cell types investigated thus far, ASNS mRNA and protein expression is rapidly increased upon Asn deprivation, as a result of the transcriptional response to the AAR (see below) but, while the fast increase in mRNA occurs also in ALL cells (22), the increase in protein is severely delayed, suggesting the existence of an active translational silencing mechanism. It is this delay in the increase of ASNS protein expression that renders ALL cells sensitive to ASNase (23). Possible translational control may explain the numerous clinical reports that showed no correlation between ASNS mRNA and ASNase sensitivity (24). Recently, Jiang et al. have demonstrated that methylation status of the *ASNS* promoter is not the same in different ALL models and that hypermethylation inversely correlates not only with the basal ASNS expression but also with the capacity to trigger the ATF4-dependent increase in ASNS expression upon following Asn depletion (25). However, although, intuitively, ASNS induction has been correlated with resistance to ASNase, it has been known for many years that ASNase-resistant ALL cells present a complex phenotype. Indeed, if ASNS overexpression is sufficient to induce the ASNase-resistant phenotype in specific ALL cell models (22), adequate availability of the ASNS substrates Gln and aspartate (Asp) requires multiple adaptation mechanisms (26, 27) (Figure 1).

The relationship between ASNS expression and ASNase sensitivity/resistance has also been complicated by the fact that both the bacterial ASNases exploited in therapy, derived from *Escherichia coli* or from *Erwinia chrysantemi* [now *Dickeya dadantii* (28)], are endowed with a low level of glutaminase activity (29). Therefore, after ASNase infusion, the depletion of both Asn and Gln ensues, although at different levels of severity and with different kinetics. The capacity of counteracting glutamine depletion is obviously also relevant for the cellular adaptation to ASNase-dependent nutritional stress. Indeed, ASNS protein induction would be functionally less effective in conditions of severe cell depletion of Gln, since human ASNS requires Gln as its obliged ammonia-donating substrate (Figure 1).

The issue of the relevance of the glutaminase activity for the antileukemic effects of ASNase has been widely debated [see for review (30)]. In the last few years, importance has been attributed to residual ASNS protein expression in ALL blasts. Chan et al. obtained a mutant *E. coli* ASNase, devoid of glutaminase activity, which is fully effective toward ASNS-null ALL blasts, but not toward ALL blasts with a residual expression of ASNS protein (31). Unfortunately, no attempt was made to correlate ASNS protein expression to enzymatic activity. However, more recent results from the same group, obtained with a murine leukemia model of ASNS-null ALL, indicate that, actually, glutaminase activity was needed for a durable suppression of the tumor (32). Further investigation is needed to fully understand the role of cellular Gln levels on ASNase sensitivity and glutaminase action in ALL progression.

## Low Expression of ASNS as a Marker of Sensitivity to ASNase in Other Cancers

The assumption that low ASNS expression represents the major hallmark for sensitivity to ASNase prompted the research of other Asn-auxotroph cancers. As far as hematological cancers are concerned, ASNase has been proposed for the therapy of several conditions [see for review (33)]. Several years ago it was demonstrated that the M5 subgroup of acute myeloid leukemias (AML) is characterized by low ASNS expression and, hence, high sensitivity to ASNase (34). A more complete attempt to categorize AML subgroups on the basis of ASNase sensitivity indicated that M1 and M0 were the most sensitive, while M3 and M7 were poorly sensitive and M4-M5 were confirmed to have a moderate sensitivity (35). Although no correlation was made between ASNase sensitivity and ASNS protein expression in that paper, a good response to therapy associated with low *ASNS* mRNA expression was later reported, at least for M0 (36). More recently, since chromosome 7 monosomy (-7) is frequently detected in adverse-risk AML and therapy-related myeloid neoplasms in children, the hypothesis that this aberration correlates with sensitivity to ASNase was investigated (37). Monosomic cells were indeed more sensitive to ASNase and exhibited significantly lowered ASNS mRNA and protein expression (37). However, the correlation between ASNase-sensitivity and ASNS expression of AML was not considered strong (33), consistently with the importance attributed to Gln, rather than Asn depletion, in the mechanism of the cytotoxic effects of bacterial ASNases on AML cells (38, 39).

ASNase has greatly improved the therapy of Natural Killer (NK)/T cell lymphoma, an aggressive lymphoid tumor associated with a poor prognosis (40, 41). Using a panel of 7 lymphoma cell lines and a retrospective analysis of patient samples, Li et al. demonstrated that ASNS expression inversely correlated with sensitivity to ASNase and positive clinical outcome (42). These data have been substantially confirmed in a more recent study (43).

ASNS has been investigated in solid tumors for many years, and the emerging picture is quite complex. In more than 50% of sporadic pancreatic ductal adenocarcinomas (PDAC), ASNS protein expression is very low (44), an observation that should be considered in light of the fact that normal exocrine pancreas has the highest basal ASNS expression of any tissue in the body (8, 45). Moreover, pancreatitis is one of the primary clinical complications exhibited by ALL patients treated with ASNase (46), suggesting that pancreatic exocrine cells are particularly sensitive to Asn depletion. Consistent with low ASNS expression, PDAC cell lines were sensitive to ASNase, and the most sensitive expressed the lowest levels of ASNS (44). Furthermore, in pancreatic cancer cells, ASNS induction is caused by glucose deprivation and is associated with increased resistance to cisplatin-induced apoptosis (47). Collectively, these data support the possible exploitation of ASNase in selected cases of low-ASNS PDAC, although they must also be interpreted in the context of the complex metabolic peculiarities of pancreatic cancer (48). Recently, it has been demonstrated that *ASNS* hypermethylation leads to the lack of ASNS protein expression in gastric and liver cancer cells, making them sensitive to *E. coli* ASNase treatment both *in vitro* and *in vivo* (49). Thus, patients could be stratified for ASNase trials on the basis of ASNS protein expression level.

## High ASNS Expression in Cancer: a Pro-Tumor Enzyme?

In several models of human solid cancers ASNS expression has been found to be positively correlated with tumor growth and, in some cases, chemo-resistance, especially if cis-platinum-derived drugs are involved (47, 50, 51). Interestingly, a recent report indicates that ASNS may be itself an additional target of platinum(II) compounds, and that these drugs cause a decrease in cell Asn as a consequence of ASNS inhibition (52). However, independently of effects on chemoresistance, ASNS overexpression has been linked to unfavorable clinical outcomes in multiple cancers (53). For example, as a possible extension of ASNase exploitation to solid tumors, Lorenzi et al. reported that the sensitivity to ASNase of cell lines derived from human ovarian carcinomas was inversely correlated with ASNS mRNA abundance (54) and, even more strongly, with ASNS protein levels (55). In other cases, direct genetic targeting of ASNS expression has been used to document enzyme effects on cancer cells. For instance, ASNS silencing lowers proliferation of human gastric cancer cells either *in vitro* or *in vivo* and synergizes the cytotoxicity of cisplatin (50). ASNS mRNA is significantly overexpressed in human gastric cancer samples compared with normal gastric tissue, and its expression inversely correlates with patient survival (50). ASNS knockdown hinders growth of melanoma cells and epidermoid carcinoma cells, inducing cell cycle, down-regulation of CDK4, CDK6, and Cyclin D1, and induction of p21^WAF^ (56). With a similar approach, Xu et al. reported a role for ASNS in the growth and colony formation ability of lung cancer (NSCLC) cells and demonstrated higher ASNS expression in lung cancer tissues than in normal lung tissue (57). A pharmacological approach was instead adopted by Hettmer et al. who demonstrated that an adenylated ASNS inhibitor inhibits the growth of murine and human sarcoma cell lines (58). In the same contribution, ASNS silencing lowered the portion of cells in S phase, an effect rescued by exogenous asparagine, and ASNS expression was found in a substantial portion of human rhabdomyosarcomas (over 70%) and in a smaller, but significant percentage of human leiomyosarcomas (more than 40%).

In breast cancer cells, ASNS is a target of IGF1/IGF2-dependent anabolic signaling (59), and, consistently, ASNS silencing depressed cell proliferation in two distinct cell lines, one of which derives from a triple negative tumor (60). Moreover, ASNS expression and Asn availability have been found to be strongly correlated with the metastatic behavior of breast cancer (61). Interestingly, in this study ASNS knock down did not affect the growth of the primary tumor but its metastatic behavior, which was significantly promoted, together with epithelial-to-mesenchymal transition, by enforced ASNS expression (61). From xenografts of the triple negative breast cancer cell line MDA-MB-231 Ameri et al. (62) obtained circulating tumor cells (CTC), which exhibit an increased capability of inducing ATF3 and ATF4 under hypoxic conditions, higher ASNS expression and a more aggressive phenotype *in vitro* and *in vivo*. Mining publicly available datasets, Lin et al. demonstrated that, among the breast cancer subtypes, triple negative has the highest ASNS protein expression (53).

As far as prostate cancer is concerned, data on possible derangements of ASNS expression in cells derived from this tumor have been known since several years. *ASNS* was included in a group of over-expressed genes in prostate cancer cells adapted to grow in suspension (63). More recently, *ASNS* mRNA overexpression, due to increased copy number of the gene, was detected in surgical specimens of castration-resistant prostate cancer and correlated with ASNS protein abundance (64). Moreover, ASNS protein expression was associated with progression to a therapy-resistant disease state (64). Interestingly, the effects of ASNase on the PC3 prostate cancer cell line and ASNS induction have been used to validate a detection system for measuring restrictive amino acids in tumors based on ribosome profiling (diricore, a procedure for **di**fferential **ri**bosome measurements of **co**don **re**ading (65).

Somewhat contradictory findings have been obtained for the role of ASNS in human hepatocellular carcinoma (HCC). Indeed, although ASNS was overexpressed in HCC, low expression has been found to be a negative outcome marker, at least in terms of overall survival, and experiments with HCC cell lines indicated that ASNS hinders cell proliferation, migration, and tumorigenicity (66). On the contrary, Li and Dong have reported that ASNS levels, along with those of the ER stress-related transcription factor ATF6, are lower in HCC than in either control subjects or patients affected by chronic hepatitis B (67). While the reasons for the discrepancy between these two studies are unclear, it should be noted that Zhang et al. studied ASNS protein expression (66), whereas only mRNA was measured by Li and Dong (67). As noted above for ALL and ovarian cancer, there can be a lack of correlation between ASNS mRNA and protein expression. Interestingly, Li and Dong discovered an *ASNS* polymorphism (rs34050735), corresponding to the 5' UTR region of the mRNA, that was significantly associated with HCC (67).

In colorectal cancer ASNS expression may also have pro-tumor or anti-tumor roles. ASNS has been found up-regulated in several human cell lines and clinical specimens derived from colon carcinoma with mutated *KRAS* (68). In particular, in a series of 93 patients, ASNS protein was high in over 70% of the *KRAS*-mutated cases but only in 30% of those with wild-type *KRAS*. ASNS expression was induced by KRAS-activated signaling, in particular through the PI3K-AKT-mTOR pathway, and repressed upon *KRAS*-silencing. Moreover, *ASNS* knockdown *in vivo* suppressed the growth of *KRAS*-mutant colon cancers, suggesting a tumor-favoring role of the enzyme in these cancers (68). However, the situation *in vivo* may be more complex. Indeed, Lin et al. have reported that low ASNS expression could constitute a negative prognostic factor, using both transcriptional data from public databases and immunocytochemical analysis of a cohort of 172 patients. In particular, ASNS low expression was significantly associated with advanced post-treatment tumor, nodal status, inferior tumor regression grade, shorter local recurrence-free survival, metastasis-free survival and disease-specific survival, and was predictive of worse outcomes and poor therapeutic response to neo-adjuvant therapy (69). It is tempting to attribute these discrepancies to differences in the mutational status of the tumors but further data are needed to confirm this hypothesis.

## ASNS in Cancer: Beyond Asn Synthesis?

A direct link between the effects of ASNS expression on cancer cells and Asn production has been recently demonstrated. Looking for regulators of the metastatic behavior in human colon cancer, Duquet et al. (70) identified the sex-determining region Y (SRY)-box, member 12 (*SOX12*), as a suppressor of metastatic behavior of HT29 xenografts. However, in a more recent paper, Du et al. (71) demonstrate that in two independent, large colorectal cancer cohorts HIF-1α-mediated *SOX12* overexpression is not only associated with a metastatic behavior, but also with a poor prognosis. Moreover, it also enhanced cell proliferation *in vitro*. Investigating the underlying mechanisms, they discovered that Asn synthesis was greatly favored by *SOX12* through the coordinated induction of glutaminase, glutamic oxaloacetic transaminase 2, and *ASNS*. These observations were confirmed in samples from patients. Consistent with the observations in the patients, down-regulation or overexpression of the three enzymes had opposite effects on cell proliferation and metastasis development. The role of Asn in these effects was confirmed by the inhibition of tumor growth and metastasis by ASNase (71).

However, although it is tempting to attribute ASNS effects on cancer growth to the Asn synthesizing activity of ASNS, its role may not be simply ensuring Asn availability for protein synthesis. In this case, very small amounts of Asn would be sufficient for cell viability and growth. Moreover, increased ASNS activity may not produce large effects on the intracellular levels of Asn, since Asn exerts a product inhibition on the enzyme acting on the recognition site for glutamine.

In fact, Asn could have additional roles, as recently suggested by Krall et al. (72), who demonstrated that Asn, either produced by the cell through ASNS activity or imported from the medium, is used as an exchange factor to promote entry and consumption of other amino acids, such as serine, arginine and histidine, and consequently, activate mTORC1 activity and protein synthesis. The transport routes responsible for the exchange were not identified, although the authors suggest that the ubiquitous exchange transporter for neutral amino acids LAT1 may be involved (72). However, earlier characterization work on LAT transporters would instead suggest that LAT2, rather than LAT1, mediates the efflux of amino acids with amido-side chain, such as Gln and Asn (73, 74). Moreover, as a determinant of serine uptake, Asn may modulate both serine metabolism and nucleotide synthesis (72).

Recent results would indicate that the relationships between ASNS, Asn, and mTORC1 activity may be more complex than envisaged. Indeed, ASNS silencing in melanoma and colon carcinoma cells causes the activation of the MAPK cascade and the activation of mTORC1 that, in turn, potentiates ATF4-dependent ASNS induction (11). Under the conditions adopted by Pathria et al., intracellular Asn is lowered by 30% by ASNS silencing, but the hypothesis that this decrease accounts for MAPK and mTORC1 activation was not directly verified, leaving open the question if these effects are due to changes in intracellular Asn or to some other ASNS-dependent mechanisms. However, these important contributions provide insight into how Asn can influence protein synthesis and cell viability well-beyond its role of proteinogenic amino acid, explaining why, in some instances, it can compensate for Gln starvation (75).

ASNS may also have other roles in cancer cell metabolism, possibly related to its participation in the response to cell stress. In NSCLC, for example, KRAS promotes ATF4 pathway activation during nutrient depletion, promoting amino acid uptake, and Asn biosynthesis (76). In the same cell model, ASNS contributes to apoptotic suppression, protein biosynthesis, and mTORC1 activation, while ASNS repression due to the inhibition of AKT had an anti-tumor effect, which is enhanced by the depletion of extracellular Asn (76). KRAS-mediated overexpression of ASNS has been also described in colon cancer in the context of adaptation to nutritional stress upon Gln starvation (68). That study found that mutated KRAS caused Asp decrease and Asn increase and that these changes were associated, both in cancer cell lines and primary tumors, with increased ASNS expression through the PI3K-AKT-mTOR pathway. These cells were resistant to Gln depletion, a behavior suppressed by ASNS knockdown but rescued if ASNS-silenced cells are incubated in Asn-supplemented medium. Moreover, both ASNS knock-down and the combined treatment with rapamycin and ASNase inhibited the growth of KRAS-mutant colon cancer xenografts *in vivo* (68). The relationship between KRAS mutations and ASNS expression may underlie a specific role of Asn in autophagy regulation. It is known that the knockout of *Atg5*, a gene needed for the autophagic response, significantly extends the survival of a murine model of salivary duct carcinoma (SDC) driven by oncogenic KRAS^G12V^, while it causes a specific Asn deficiency and a compensatory ASNS overexpression (53). Consistently, autophagy or ASNS inhibition reduced KRAS-driven tumor cell proliferation, migration, and invasion, all effects rescued by Asn supplementation. Finally, these observations were reflected in human cancer-derived data, linking ASNS expression and malignancy (53).

A role for ASNS in the cell response to nutritional stress has been also shown by Ye et al. (77), considering that its master activator ATF4 is also overexpressed in human tumors. Overexpression of ASNS or Asn supplementation, but not of other non-essential amino acids, counteracts the proliferative block and cytotoxicity due to ATF4 silencing in human fibrosarcoma and colorectal adenocarcinoma cells. The knockdown of ATF4, or the suppression of its induction by GCN2 silencing, inhibited tumor growth *in vivo* (77). These results have been further extended by Tameire et al. (78), demonstrating that MYC upregulates ATF4 through GCN2 activation and that, subsequently, ATF4 induces several genes that are also MYC targets, many of which involved in amino acid transport (such as *SLC1A5*) and metabolism (such as *ASNS*). This group of genes also includes *4E-BP1*, leading the authors to hypothesize that, through ATF4-mediated gene induction, tumor cells couple enhanced translation rates with survival. In several human tumors, such as diffuse large B-cell lymphoma, colorectal cancer, breast cancer and sarcoma, 4E-BP1 levels were positively correlated with ATF4-target genes, including *ASNS* (78). These results potentially link ASNS induction and the successful response to oncogene-dependent proteotoxic stress and hence cancer cell survival, although the precise role played by ASNS in these complex mechanisms awaits further investigation.

## ASNS Expression as a Metabolic Vulnerability

Together with Asn auxotrophy, associated with ASNS silencing, arginine auxotrophy, which depends on absent expression of argininosuccinate synthase 1 (ASS1), represents another, widely investigated metabolic vulnerability in human cancers. In arginine-auxotroph human breast cancer cell lines, arginine depletion induces ASNS, provoking a depletion of Asp that hinders malate-aspartate shuttle and promotes cell death (79). Thus, in this particular model, ASNS induction, rather than constituting a pro-survival mechanism, would promote cytotoxicity through Asp depletion.

The importance of Asp metabolism in cancer has been increasingly recognized in the last few years (80–82). Since human cells cannot use Asn as a source of Asp, due to lack of sizable expression of enzymes with asparaginase activity, the metabolic relationship between the two amino acids is a one-way pathway, where Asp can be used a Asn source, while the reverse is not possible. Interestingly, if the expression of guinea pig ASNase is forced in human cancer cells, Asn uptake can fuel the intracellular pool of Asp, and cell growth is stimulated (83), providing a proof-of-principle demonstration of the importance of an adequate Asp availability for fast cell proliferation. Membrane transport can limit cell availability of Asp, which, at the levels present in human plasma, relies on the activity of high-affinity, sodium-dependent EAAT transporters (84, 85). A member of the family, EAAT1, coded by *SLC1A3*, has been identified as an important contributor of resistance to ASNase in several lines of prostate cancer cells (27). In one of these models, although *ASNS* is heavily induced upon ASNase treatment, cell death is not prevented if EAAT1 is pharmacologically inhibited (27). Interestingly, prostate cancer cell lines endowed with low expression of EAAT1 exhibit sizable levels of other EAATs, such the ubiquitous EAAT3, which is regulated at transcriptional level under various stress conditions (85).

Another example of possible ASNS-mediated vulnerability comes from the studies of Wong et al. on KRAS-mutated colorectal cancers. *SLC25A22*, which encodes a mitochondrial glutamate transporter, is one of the genes up-regulated in these tumors. Increased SLC25A22 protein was observed in colorectal cancer tissues and was associated with shorter survival, while transporter knock-down hindered cancer cell proliferation, migration, invasion *in vitro* and tumor formation and metastasis *in vivo*. The biochemical alteration attributable to *SLC25A22* knockdown and accounting for the anti-proliferative effects is the inhibition of Asp biosynthesis and the consequent depletion of oxaloacetate leading to hampered regeneration of NAD^+^ and NADP^+^, glycolysis hindrance and energetic crisis. In this context, the inhibition of ASNS-mediated Asn synthesis would be another effect of Asp depletion, specifically leading to hindered cell migration (86). One would wonder what are the links between the two KRAS-mediated effects on Asn-Asp metabolism. Although SLC25A22 would be permissive for Asp synthesis, ASNS induction would promote its consumption, suggesting that a dysregulated ASNS expression would be, in fact, a menace for the energetic equilibrium of the cancer cell.

## Discussion

The examples discussed in the last paragraph indicate that, in some cancers, low ASNS expression may be advantageous. However, most of the epidemiological and experimental evidence gathered thus far suggest a pro-cancer role of the enzyme, pointing to a metabolic advantage for high-ASNS cancer cells. Thus, both low- and high-ASNS expression may imply metabolic advantages in particular cancer models (Figure 1). It should be remarked that either situation also implies some potential metabolic vulnerabilities, such as Asp depletion, for high-ASNS tumors, and Asn auxotrophy, for low-ASNS cancers.

**Figure 2:**
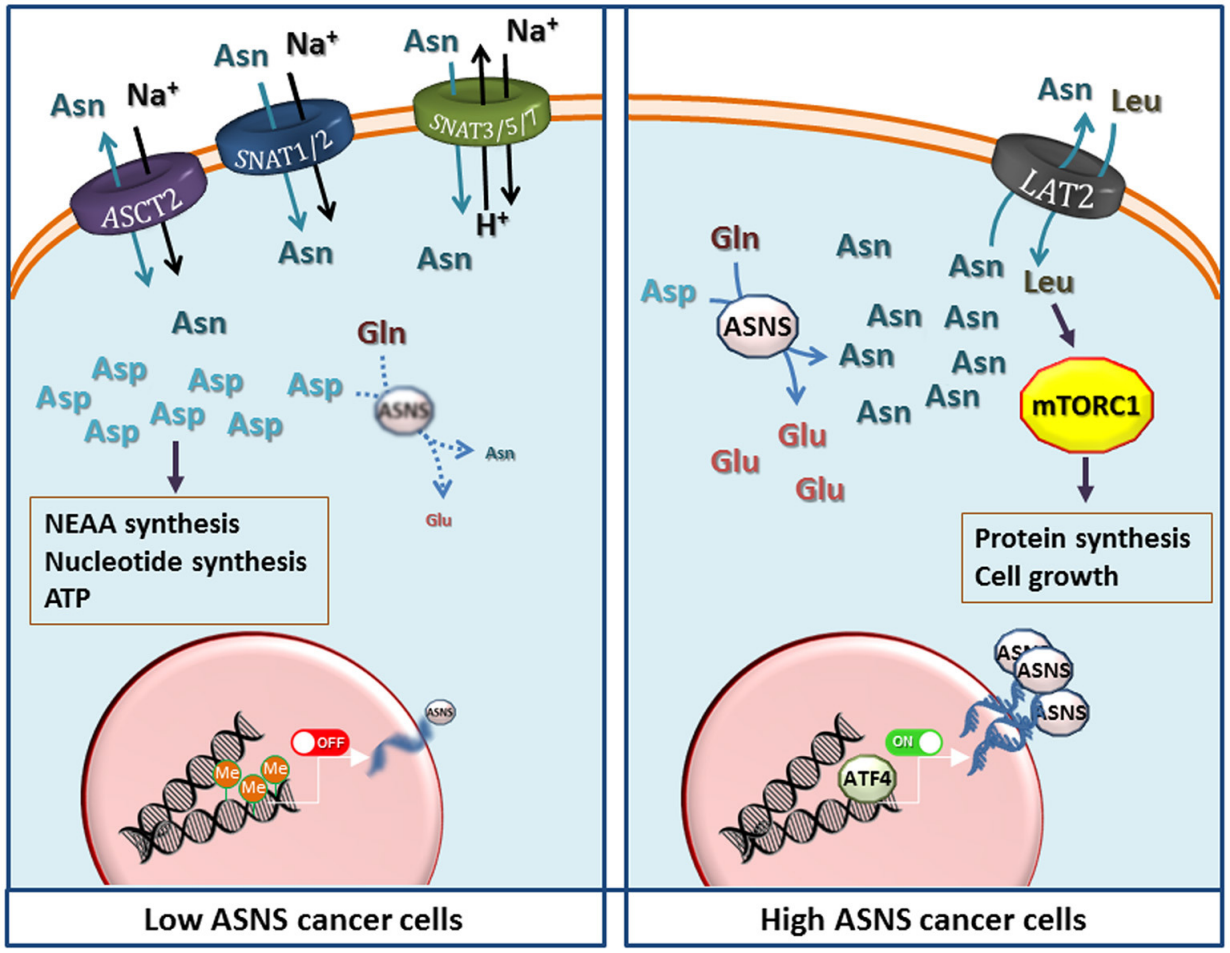
The potential metabolic advantages of low and high expression of Asparagine Synthetase (ASNS) in cancer cells. (Left) Low ASNS expression, caused by promoter methylation, renders cells dependent on asparagine (Asn) uptake, while increasing aspartate (Asp) availability for the synthesis of nucleotides, other non-essential amino acids (NEAA), and energy production. Transporters most likely involved in Asn uptake are shown, although the indication is largely hypothetical in most cancer models. (Right) Increased *ASNS* transcription, due to either gene amplification or high ATF4 activity, raises Asn production that enhances protein synthesis and cell growth by activating the mammalian target of rapamycin complex 1 (mTORC1) through the influx of essential amino acids mediated by exchange through a LAT transporter (72), tentatively identified as LAT2. Other transporters have been omitted for clarity. However, other mechanisms, such as direct effects of Asn or Asp on mTORC1, should not be excluded but the information available (11, 68, 72) does not allow generalizable conclusions. See text for discussion.

If high ASNS expression really confers marked metabolic advantages, one wonders what is the significance of ASNS silencing in the majority of ALL blasts and in the other examples of Asn-auxotroph tumors, discussed above. For these cancers, the maintenance of the intracellular pool of Asp seems more important than ensuring an intracellular source of Asn. In these cells, blocking the expression of ASNS would indeed leave most cell Asp available to other metabolic pathways, such as nucleotide and non-essential amino acid synthesis or energy production (Figure 2). On the other hand, Asn auxotrophy not only has the obvious consequence of an increased sensitivity to Asn depletion and, hence, to ASNase treatment, but also entails a strict dependence of the cancer cells on extracellular sources of the amino acid even under normal growth conditions. Since Asn plasma levels are much lower than those of Gln, and the transport systems for neutral amino acids are usually endowed with fairly high Km values, it is expected that Asn auxotroph tumors establish close relationships with their microenvironment to exploit neighboring cells as an efficient Asn source. Actually, metabolic support to ASNase-treated ALL blasts by ASNS-expressing mesenchymal stromal cells has been reported (87), although mechanisms underlying the putative Asn fluxes have not been investigated.

At variance with the transport of other amino acids, in particular Gln, which shares many structural similarities with Asn and is its metabolic precursor, the characteristics of Asn transport have not been extensively studied in Asn-auxotroph cancer cells. It is known that Gln, Asn and His are substrates of the so called “N system” transporters (88), such as SNAT3, SNAT5 and SNAT7 (89, 90), but little information is available on the expression of these transporters in cancer tissues, and no attempt has been made thus far to correlate their expression with that of ASNS. Also other transporters of the SLC38 family, such as the System A carriers SNAT1 and SNAT2 (90), and the product of *SLC1A5* (ASCT2) (91) accept Asn as a substrate. However, lack of a comprehensive knowledge of Asn transporters in cancer constitutes an important gap given that Asn membrane fluxes are obviously essential for the survival of Asn-auxotroph cancer cells. The definition of transport mechanisms involved in Asn transmembrane fluxes would be highly valuable also for the biology of high-ASNS cancers, which are thought to export sizable amounts of the amino acid into the extracellular medium (72). In these tumors, Asn may work as a modulator of the behavior of normal cells within the cancer microenvironment, as recently suggested for endothelial cells (92).

The results recounted in this contribution indicate that the role played by ASNS may be cancer-specific and should be assessed on an individual basis. Therefore, to better define the role of ASNS expression and activity in human cancers, specific and potent inhibitors would be extremely important and have been actively searched for many years (93, 94). Many classes of compounds have been proposed thus far (29), in some cases with high potency (95) and promising results *in vitro* (96, 97), but no specific ASNS inhibitor is yet in clinical experimentation or even commercially available. As a consequence, experimental ASNS inhibition still relies on genetic manipulation. However, most recently, Zhu et al. (7) have described a slow-onset inhibitor, which binds to a negatively charged cluster of side chains in the synthetase domain of human ASNS with nanomolar affinity and a good specificity *in vitro* and may be the basis for novel anticancer compounds targeting ASNS.

## Author Contributions

MC, GT, MB, MK, and OB drafted the manuscript and approved it.

### Conflict of Interest

The authors declare that the research was conducted in the absence of any commercial or financial relationships that could be construed as a potential conflict of interest.
